# Two horizons, one burden: estimating lifetime and decade-long medical costs of childhood obesity in Kazakhstan

**DOI:** 10.3389/fpubh.2026.1853931

**Published:** 2026-10-02

**Authors:** Berik Koichubekov, Khamida Abdikadirova, Nazgul Omarbekova, Gulzat Zhunussova, Azamat Kharin

**Affiliations:** 1 Department of Informatics and Biostatistics, Karaganda Medical University, Karaganda, Kazakhstan; 2 Department of Physiology, Karaganda Medical University, Karaganda, Kazakhstan

**Keywords:** childhood obesity, economic burden, healthcare costs, healthcare economy, Markov model

## Abstract

**Objective:**

To estimate the lifetime and cumulative direct medical burden associated with childhood overweight and obesity in Kazakhstan using a dynamic age-structured modeling approach.

**Methods:**

A dynamic age-structured Markov cohort model was developed to simulate the progression of children aged 5–9 years through successive age groups and weight states (normal weight, exclusive overweight, and obesity). Transition probabilities were reconstructed using a synthetic cohort approach based on repeated cross-sectional prevalence data and were calibrated to reproduce observed epidemiological patterns. State-specific annual direct medical costs were estimated using a normative costing approach based on national clinical protocols and tariffs. Lifetime burden was calculated as the discounted sum of future medical costs (3% discount rate). Furthermore, cumulative short-term was estimated for the period 2024–2035. Deterministic sensitivity analysis and structural scenario analysis were conducted to assess model robustness. Policy scenarios examined the impact of reducing childhood obesity prevalence by 5 and 10%.

**Results:**

The average discounted lifetime cost per child was 96,996.26 KZT (USD 206.37), resulting in a total national lifetime financial impact of 194 billion KZT (USD 413 million) for the 2024 cohort of children aged 5–9 years. Over the 2024–2035 horizon, cumulative discounted direct healthcare expenditure reached 128.72 billion KZT (USD 273.9 million), with approximately 71% attributable to obesity. Sensitivity analysis showed that model results were most responsive to parameters related to obesity prevalence and costs. Structural scenarios indicated that the base-case model likely underestimates cost, with age-varying adult costs increasing estimates by up to 13.6%. Policy scenarios demonstrated that a 5 and 10% reduction in childhood obesity prevalence could reduce lifetime burden by 4.8 and 9.4%, respectively.

**Conclusion:**

Childhood obesity imposes a substantial and persistent economic impact on Kazakhstan's healthcare system. Preventive interventions targeting obesity in early life may yield significant long-term cost savings and should be prioritized in national health policy.

## 1 Introduction

Overweight and childhood obesity have become a major public health problem worldwide in recent decades. Previously considered a problem affecting pre-dominantly high-income countries, overweight is now increasingly common in low- and middle-income countries, reflecting profound changes in eating habits, urbanization and lifestyles. According to 2020 data 20.6% of the examined children in Kazakhstan were overweight and obese (1). This epidemiological shift has important implications not only for public health, but also for the resilience of health systems.

Overweight and obesity are key risk factors for chronic non-communicable diseases such as hypertension, hyperlipidaemia, type 2 diabetes mellitus, cardiovascular disease, metabolic syndrome, high cholesterol, and cancer (2, 3). Along with an increased risk of chronic disease, obesity in children is strongly associated with mental health problems such as depression, anxiety, and low self-esteem, often exacerbated by social stigma and body dissatisfaction (4). Moreover, the clinical consequences of obesity lead to significant economic costs. These include direct health costs associated with the diagnosis, treatment and management of obesity and related diseases, as well as indirect costs such as lost productivity. At the macroeconomic level, obesity has been shown to reduce the supply of labor and economic output, placing additional pressure on public finances (5).

The economic burden of obesity continues to grow globally and may account for a significant proportion of total health expenditure in many countries (6). For example, it has been shown that in the United States the annual incremental medical costs were USD237.55 per capita attributable to childhood overweight and obesity. Overweight and obesity caused a per capita increase of USD56.52, USD14.27, USD46.38, and USD1975.06 for costs in non-hospital healthcare, outpatient visits, medication, and hospitalization, respectively (7). These costs are unevenly distributed throughout life: people who are obese from an early age tend to accumulate higher healthcare costs over the course of their lives compared to those who become overweight later in life. The review (8) notes that the relative costs of care were 35% higher and direct of care were between 0.4 and 88% higher in children with obesity than normal weight children. In absolute terms, the excess costs (USD, 2024) for treating overweight and children with obesity ranged from USD32.44 to USD1225.35. The annual per capita healthcare costs (USD) differed across countries, which was attributed to methodological differences in cost estimation and durations of the studies.

Childhood represents a critical period in the development of obesity and its long-term consequences. Studies suggest that being overweight in childhood is a strong predictor of obesity in adolescence and adulthood. Obese children are more likely to remain obese later in life and develop a range of chronic diseases, including type 2 diabetes, cardiovascular disease, and metabolic disorders (9) As a result, the health and economic consequences of childhood obesity extend well beyond the pediatric period and contribute to sustained increases in healthcare utilization over time. From a life cycle perspective, early intervention is therefore essential to prevent the accumulation of both clinical and economic impacts (10).

Despite growing recognition of the importance of childhood obesity, evidence on its economic burden remains limited, especially in emerging economies. Most of the existing studies were conducted in high-income countries and may not be applicable to other settings due to differences in health systems, cost structures, and epidemiological patterns (11). For example, the review (7) included publications on obesity lifetime burden for 2001–2021. Among the included 48 studies, 29 were conducted in the United States, seven in Europe (including five in Germany, one in Ireland, and one in the Netherlands), five in Australia, four in Canada, and three in Japan. Many countries, including Kazakhstan, lack comprehensive national estimates that reflect both the short- and life-long economic consequences of childhood overweight and obesity. This gap limits the ability of policymakers to assess the scale of the problem and prioritize effective prevention strategies.

From a methodological perspective, estimating the cost implications of childhood obesity presents several difficulties. Longitudinal data tracking individuals across the lifespan are often lacking, making it difficult to directly observe changes in weight over time (12). As a result, many studies rely on simplistic assumptions or cross-sectional analyses that do not fully reflect the dynamic nature of obesity progression. Additionally, relatively few models simultaneously take into account demographic aging, epidemiological changes and long-term cost accumulation within a single model. To overcome these limitations, modeling approaches are required that can integrate multiple data sources and reconstruct plausible lifecycle trajectories (13).

To address these problems, this study developed a dynamic age-related Markov cohort model to estimate the financial impact of overweight and obesity in children in Kazakhstan. The model combines age progression with transitions between weight states to model the evolution of a cohort of children aged 5–9 years over time. Using a synthetic cohort approach based on prevalence data from repeated cross-sectional studies, the model reconstructs the probabilities of transitions in the absence of longitudinal observations. This framework allows you to estimate both the lifetime economic burden and the short-term total healthcare costs associated with being overweight.

Likewise the study includes sensitivity analysis and structural scenario analysis to assess the sustainability of the results to key modeling assumptions. Policy scenarios are considered to assess the potential economic benefits of reducing the prevalence of childhood obesity. By providing a comprehensive assessment of both long-term and short-term economic impacts, the study aims to generate data that can inform and support national health policies.

## 2 Materials and methods

In our study, the lifetime burden represents the financial impact that childhood obesity creates for the healthcare system of Kazakhstan throughout the life course of the cohort of children, starting at the age of 5–9 years and continuing as they move into adolescent and adult age groups. This means that the lifetime burden answers questions such as the expected lifetime healthcare expenditure attributable to one child over a long follow-up period; how much spending the health system will accumulate if the current cohort of obese children moves into adulthood; what is the total present cost of this problem for the population.

### 2.1 Data collection

Data used in this study were obtained from publicly available international and national statistical sources. Information on the prevalence of exclusive overweight and obesity among children and adolescents was retrieved from the UNICEF database, which provides standardized indicators based on body mass index-for-age (BMI-for-age) definitions.

Demographic data, including population size by age groups, were obtained from the official statistics of the Bureau of National Statistics of the Republic of Kazakhstan. These data were used to estimate the size of the baseline cohort of children aged 5–9 years in 2024 and to calculate the number of individuals in each weight state.

Historical prevalence data were used to reconstruct age-specific distributions and to derive age-group tracking coefficients using a synthetic cohort approach. Specifically, prevalence observed in earlier cohorts was linked to later age groups to approximate longitudinal transitions in the absence of individual-level follow-up data.

All datasets were aggregated and de-identified; therefore, no ethical approval was required.

### 2.2 Modeling framework

A dynamic age-structured Markov cohort model was developed to estimate the lifetime medical burden attributable to childhood overweight and obesity. The model was designed to combine demographic progression across age groups with epidemiological progression across weight states. This approach made it possible to simulate the life-course trajectory of a pediatric cohort and to estimate the cumulative healthcare costs associated with excess weight from childhood into adulthood (Figure 1). For this reason, the present study employed two interconnected transition structures: age-group progressing matrices and weight-state transition matrices.

**Figure 1:**
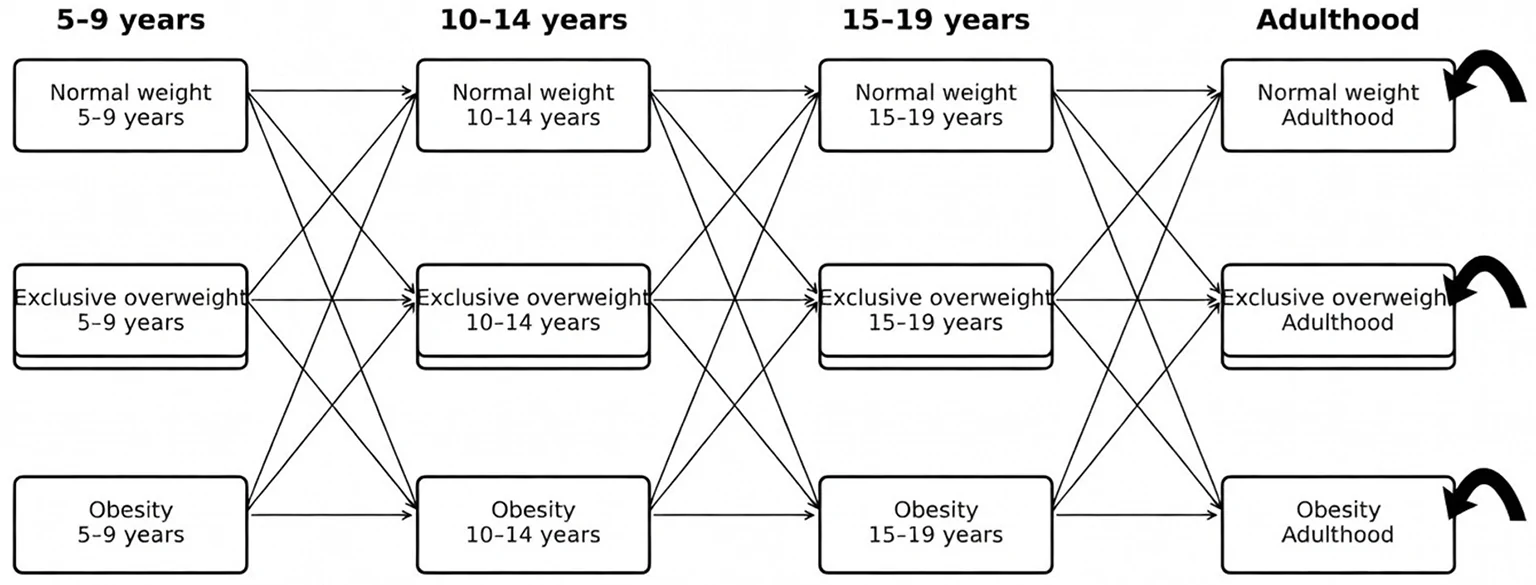
Age-structured Markov model for childhood overweight and obesity progression.

Age progression in the model was represented deterministically as cohort aging. Individuals in the 5–9 years age group progressed to the 10–14 years group, then to the 15–19 years group, and subsequently to adulthood. Thus, age transitions reflected demographic progression of the cohort rather than probabilistic processes. Age progression was represented by an age-transition matrix *A*, such that the vector of population counts across age groups at time *t* + 1 was given by:


$$\begin{array}{l} {\text{n}}_{t+1}={\text{n}}_{t}A \end{array}$$ (1)

where **n**_*t*_ is the vector of age-specific population counts at time *t*.

Weight-state transition matrices were introduced to model changes in body weight status over time. These matrices reflect the epidemiological dynamics of the condition by describing the probability that an individual in a given year will remain in the same weight category or move into another category. In the present framework, the relevant states are normal weight, exclusive overweight, and obesity. Because longitudinal cohort data describing individual transitions between weight states are not available for Kazakhstan, transition probabilities were estimated using a synthetic cohort approach based on repeated cross-sectional prevalence data. Transition matrices were calibrated so that the model reproduced the observed prevalence of weight states in the subsequent age group. Where multiple solutions were possible, additional constraints were applied using longitudinal evidence on weight-status tracking from the literature (14, 15). Similar calibration approaches have been applied in previous economic models of obesity resource utilization (16, 17).

For each age group and year, a complete vector of distribution by state was formed:


$$\begin{array}{l} P_{a,t}=\left[P_{N,a,t},P_{OW,a,t},P_{OB,a,t}\right] \end{array}$$ (2)

where

*P*_*N,a,t*_ is prevalence of normal body weight in the age group *a* per year *t*;

*P*_*OW,a,t*_ is prevalence of exclusive overweight;

*P*_*OB,a,t*_ is prevalence of obesity.

### 2.3 Integration of age progression and weight-state progression

The combined transition matrix *P*_*a*_ was analytically derived from age-specific prevalence distributions by solving the system


$$\begin{array}{l} \pi_{a+1}=\pi_{a}P_{a} \end{array}$$ (3)

under epidemiologically plausible structural constraints:

• sum of row probabilities = 1

• some transitions are prohibited (e.g. Normal → Obesity)

• epidemiologically plausible weight trajectories.

After imposing structural constraints on incident and remission transitions, the remaining transition probabilities were obtained by solving the resulting system of linear equations linking prevalence distributions across age groups.

### 2.4 Internal consistency and epidemiological plausibility of transition matrices

To verify the mathematical consistency of the reconstructed transition matrices, we assessed their ability to reproduce the observed prevalence distributions across consecutive age groups. Given the absence of longitudinal individual-level data in Kazakhstan, transition probabilities between weight states were estimated indirectly using a synthetic cohort approach based on repeated cross-sectional prevalence data. Under this framework, the observed prevalence in successive age groups is interpreted as an approximation of the trajectory of the same cohort as it ages.

Let π_*a*_ denote the vector of observed prevalence of weight states in age group *a*, and let *P*__*a*_ → *b*_ denotes the transition matrix linking age group *a* to the subsequent age group *b*. The model-implied prevalence vector in the next age group is given by:


$$\begin{array}{l} {\hat{\pi }}_{b}=\pi_{a}P_{a\to b} \end{array}$$ (4)

Transition matrices were derived by solving a system of linear equations linking prevalence distributions across age groups, subject to a set of epidemiologically and mathematically motivated constraints. These constraints included: non-negativity of all transition probabilities; row sums equal to one; and exclusion of non-identifiable or implausible transitions (e.g., direct transitions from normal weight to obesity).

Because the transition probabilities were derived analytically by solving a system of equations defined by the observed prevalence distributions, the model reproduces the empirical prevalence in subsequent age groups exactly, yielding a zero discrepancy between observed and model-predicted values. It is important to emphasize that this exact correspondence does not represent predictive accuracy or external validation, but rather confirms the internal consistency of the reconstruction procedure. The purpose of this analysis is to verify that the estimated transition matrices constitute a mathematically admissible solution that is fully consistent with the available aggregate data.

In addition to numerical consistency, the transition matrices were evaluated for epidemiological plausibility. Specifically, we verified that:

- the persistence of obesity across age groups (i.e., the probability of remaining in the obesity state) is higher than the probability of transitioning from obesity to normal weight, consistent with evidence from longitudinal studies;
- all transition probabilities are within the acceptable range of 0 to 1;
- row sums equal one.

### 2.5 Estimating the economic burden of obesity

#### 2.5.1 Assignment of state-specific costs

The analysis focuses exclusively on direct medical costs associated with exclusive overweight and obesity. We did not include indirect costs (e.g., productivity losses due to absenteeism, presenteeism, disability, or pre-mature mortality), quality-of-life impacts, or broader societal costs. This approach was chosen to provide a transparent estimate financial impact on the healthcare system, based on available national data.

To estimate the economic burden, we assigned each model state an annual direct medical cost. Costs were estimated using a normative costing approach based on national clinical protocols and relevant tariffs for outpatient care, laboratory investigations, diagnostic procedures, and follow-up management. Importantly, these estimates represent the total direct medical costs for managing patients in each weight state, rather than incremental costs relative to normal-weight individuals. This means that the costs reflect the full package of healthcare services recommended for patients with overweight or obesity, as specified in the national clinical protocols. We adopted this approach because the clinical protocols for normal-weight children do not include routine diagnostic and monitoring procedures, making the estimation of incremental costs methodologically challenging and potentially underestimating the true system burden.

Tariffs were obtained from the official schedule approved by the Ministry of Health of the Republic of Kazakhstan (Order No. 12 of April 5, 2024, About amendments to the order of the Acting Minister of Health of the Republic of Kazakhstan dated October 30, 2020 No. KP Д M-170/2020 “On approval of tariffs for medical services provided within the framework of the guaranteed volume of free medical care and in the compulsory social health insurance system”). All costs were calculated in 2024 prices and are expressed in Kazakhstan tenge (KZT). No inflation adjustments were applied, as all unit costs were expressed in constant prices of the same base year. The costs represent payer costs from the healthcare system perspective, reflecting government expenditures on medical services, excluding provider overhead and administrative costs. For international comparability, selected results were also converted to US dollars (USD) using the average exchange rate for 2024–470 KZT per 1 USD. Given the high volatility of the KZT exchange rate and the fact that healthcare costs and public expenditures in Kazakhstan are not directly indexed to USD, all primary analyses and interpretations are based on KZT values. Detailed costing assumptions, including service frequencies and unit tariffs, are provided in Supplementary Appendix A.

For each state *s* (normal weight, exclusive overweight, and obesity) the annual cost was calculated as:


$$\begin{array}{l} C_{s}=\underset{i}{\sum }f_{s,i}\cdot c_{s,i} \end{array}$$ (5)

where

*C*_*s*_ is the annual cost associated with state *s*,

*f*_*s,i*_ is the frequency of use of service *i* in state *s*,

*c*_*s,i*_ is the corresponding unit cost (tariff).

For services provided only to a proportion of patients, expected costs were calculated probabilistically:


$$\begin{array}{l} C_{s}=\underset{i}{\sum }p_{s,i}\cdot f_{s,i}\cdot c_{s,i} \end{array}$$ (6)

where *p*_*s,i*_ is the probability of receiving service *i* in state *s*.

This procedure enabled the estimation of separate annual costs for childhood exclusive overweight, childhood obesity, adult exclusive overweight, and adult obesity.

#### 2.5.2 Estimation of annual direct medical burden

For each model cycle, the total direct medical burden was estimated by multiplying the number of individuals in each state by the corresponding annual cost and summing across all states:


$$\begin{array}{l} B_{t}=\underset{s}{\sum }N_{s,t}\cdot C_{s} \end{array}$$ (7)

where

*B*_*t*_ is the total direct medical burden in year *t*,

*N*_*s,t*_ is the number of individuals in state *s*at time *t*,

*C*_*s*_ is the annual cost associated with state *s*.

This measure represents the annual direct medical cost attributable to excess weight at the population level.

#### 2.5.3 Estimation of discounted lifetime cost per child

Discounted lifetime direct medical cost per child was estimated using an age-structured Markov model describing transitions between weight states (normal weight, exclusive overweight, and obesity) over the life course.

The model followed a cohort of children aged 5–9 years. At each annual cycle, transition probabilities were applied to estimate the distribution of individuals across weight states, accounting for age progression.

The expected annual cost at time *t*was calculated as:


$$\begin{array}{l} E\left(C_{t}\right)=p_{N,t}\cdot C_{N}+p_{OW,t}\cdot C_{OW}+p_{OB,t}\cdot C_{OB} \end{array}$$ (8)

where

*p*_*N,t*_*, p*_*OW,t*_*, p*_*OB,t*_, denote the probabilities of being in normal weight, exclusive overweight, and obesity states at time *t*, respectively,

*C*_*N*_*, C*_*OW*_*, C*_*OB*_ represents the corresponding annual costs.

Lifetime cost per child was calculated as the discounted sum of expected annual costs over the model horizon:


$$\begin{array}{l} LC=\overset{T}{\underset{t=0}{\sum }}\frac{E\left(C_{t}\right)}{{\left(1+r\right)}^{t}\;} \end{array}$$ (9)

where

*LC* is the lifetime cost per child,

*T* is the lifetime horizon,

*r* is the annual discount rate (3%).

This measure represents the present value of all future direct medical costs attributable to excess weight for one child. Discounting was applied to express future healthcare costs in present value terms, reflecting time preference and allowing comparability across years. This is a standard approach in economic evaluations to account for time preference.

The model does not include mortality (obesity-related or competing) or disease progression beyond weight-state transitions. Consequently, the estimated lifetime costs reflect healthcare expenditures associated with weight states, without accounting for the effects of pre-mature mortality or the development of obesity-related complications.

#### 2.5.4 Estimation of total lifetime burden

In addition to per-child estimates, the model calculated the total lifetime burden at the population level. Total lifetime burden was defined as the aggregated discounted cost for the entire cohort:


$$\begin{array}{l} LB=LC\cdot N \end{array}$$ (10)

where

*LB* is the total lifetime burden,

*LC* is the lifetime cost per child,

*N* is the size of the initial cohort.

This measure represents the total discounted direct medical cost attributable to excess weight for all children in the cohort.

Lifetime cost reflects the average discounted direct medical expenditure per child over the life course, whereas lifetime burden represents the corresponding aggregate cost at the population level. Both measures are expressed in present value terms and do not represent projections of actual future expenditures by calendar year.

#### 2.5.5 Estimation the short-term cumulative economic burden

To estimate the short-term cumulative economic burden, annual direct medical costs attributable to childhood exclusive overweight and obesity were first calculated for the baseline year 2024. These estimates were derived using age-specific prevalence of weight states and the corresponding annual state-specific medical costs.

For projection purposes, the prevalence of exclusive overweight and obesity among children aged 5–9 years was assumed to remain constant at the level observed in 2024. Annual direct medical burden was therefore assumed to remain constant in real terms over the projection period.

The cumulative discounted burden was then calculated for the period 2024–2035 using the same discount rate applied in the lifetime analysis (3%). For each year t, the discounted annual cost was calculated as:


$$\begin{array}{l} C_{t}=\frac{C_{2024}}{{\left(1+r\right)}^{t}\;} \end{array}$$ (11)

where

*C*_2024_ is the annual direct medical burden estimated for 2024 and *r* is the annual discount rate (3%).

The cumulative burden for the period 2024–2035 was obtained as the sum of discounted annual costs:


$$\begin{array}{l} Cumulative\;burden=\overset{11}{\underset{t=0}{\sum }}\frac{C_{2024}}{{\left(1+r\right)}^{t}\;} \end{array}$$ (12)

Separate estimates were produced for:

- exclusive childhood overweight
- childhood obesity
- total burden.

### 2.6 Deterministic one-way sensitivity analysis

Sensitivity analyses were conducted to assess the robustness of model outcomes to uncertainty in key parameters. One-way sensitivity analysis was performed by varying transition probabilities between weight states, annual medical costs, childhood obesity prevalence, and the discount rate. For each scenario, lifetime medical costs per child and the total national economic burden were recalculated.

In the univariate deterministic sensitivity analysis, each key parameter of the model was changed one at a time, while all other parameters were kept at the baseline level. The base year was set to be 2024.

The following parameters were included in the analysis:

- baseline prevalence of exclusive overweight: ±20%;
- baseline prevalence of obesity: ±20%;
- discounted lifetime cost per child with exclusive overweight: ±20%;
- discounted lifetime cost per child with obesity: ±20%;
- cohort size of children aged 5–9 years: ±10%.

### 2.7 Scenario analysis

#### 2.7.1 Structural scenario analysis

Structural scenarios were designed to test whether the estimated lifetime healthcare costs of childhood overweight and obesity depends on specific assumptions regarding weight-state transitions and the specification of adult medical costs.

Three alternative model specifications were considered.

##### 2.7.1.1 Scenario A (base case).

In the base-case scenario, several transition pathways were restricted to ensure model identifiability given the use of aggregated prevalence data. In particular, incident transitions from normal weight to overweight and obesity were set to zero, as these could not be reliably estimated. Similarly, direct transitions from obesity to normal weight were not allowed. This specification was adopted as a necessary constraint to ensure model identifiability given the use of aggregated prevalence data, which does not allow for the identification of incident transitions from normal weight. Consequently, this assumption yields a conservative, lower-bound estimate of the economic impact, as it excludes trajectories in which children with normal weight develop excess weight during later childhood or adolescence. To quantify the potential magnitude of this underestimation, Scenario B was designed to relax this restriction.

##### 2.7.1.2 Scenario B (less restrictive transition structure).

In this scenario, the restriction on incident transitions from normal weight was relaxed. Small transition probabilities from normal weight to exclusive overweight and obesity were introduced, provided that the resulting transition matrix remained consistent with the observed age-specific prevalence distributions. This scenario reflects a more realistic epidemiological trajectory in which some children develop excess weight during aging.

##### 2.7.1.3 Scenario C (age-varying adult costs).

In the basic specification of the model, the annual direct medical costs for adult exclusive overweight and adult obesity were assumed to be constant for all ages. However, numerous studies show that obesity-related medical costs increase with age due to the accumulation of chronic diseases and the increase in healthcare consumption.

In the age-varying adult costs scenario, baseline estimates of annual costs for adult conditions were adjusted using age coefficients reflecting the relative increase in medical costs with age. Baseline cost levels remained tied to Kazakhstani estimates, and age dynamics were introduced through multiplicative coefficients.

Taking into account international experience in estimating age-related cost dynamics (18, 19), the following relative obesity-associated cost factors for the adult population of Kazakhstan were calculated (Table 1). The unit (1.0) is the average annual cost of an obese patient aged 20–34 years. The proposed coefficients are an integrated estimate designed to model the lifetime burden, and can be updated when direct data on age-related costs of obesity in the Republic of Kazakhstan become available.

**Table 1:** Age-related cost factor.

Age group	Cost multiplier
20–34 years	1.00
35–49 years	1.30
50–64 years	1.70
≥65 years	2.20

For each scenario, the model recalculated discounted lifetime cost per child and the total discounted national lifetime medical costs for the baseline cohort of children aged 5–9 years in 2024.

#### 2.7.2 Policy scenario analysis

To evaluate the potential economic benefits of hypothetically reducing childhood obesity prevalence, we conducted a policy scenario analysis in addition to the base-case model. It is important to emphasize that these scenarios are hypothetical modeling exercises designed to illustrate the potential magnitude of cost savings that could be achieved if childhood obesity prevalence were reduced, rather than empirical estimates of the effectiveness of specific interventions. Two alternative policy scenarios were considered:

- Scenario 1%−5% reduction in childhood obesity prevalence. In this scenario, the prevalence of childhood obesity among children aged 5–9 years was assumed to decrease by 5% relative to the baseline level. The reduction was assumed to shift children from obesity to normal weight state (conservative assumption). This scenario represents the potential effect of moderately effective prevention strategies, such as improvements in school nutrition policies, increased physical activity programs, and public health education initiatives.
- Scenario 2%−10% reduction in childhood obesity prevalence. In this scenario, the prevalence of childhood obesity was assumed to decrease by 10% relative to the baseline level. This scenario represents the potential impact of more comprehensive and sustained prevention strategies, including broader public health interventions and regulatory measures aimed at reducing exposure to obesogenic environments.

In both policy scenarios, the reduction in childhood obesity prevalence was applied to the baseline cohort of children aged 5–9 years in 2024. All other model parameters, including transition probabilities, annual medical costs, and the discount rate, remained unchanged. The model recalculated the discounted lifetime cost per child and the total national lifetime economic burden associated with childhood overweight and obesity.

## 3 Results

In the base-case model, the prevalence of childhood overweight and obesity was assumed to remain constant at the baseline 2024 level. This steady-state assumption is commonly used in burden-of-disease models to estimate the long-term economic impact associated with current prevalence levels rather than projecting future epidemiological trends.

Table 2 provides the baseline prevalence distribution of weight states by age group. These prevalence estimates serve three purposes in the model: defining the initial state distribution for the baseline cohort, enabling reconstruction of transition probabilities across consecutive age groups, and determining the population counts used in the estimation of annual and lifetime healthcare expenditure. Prevalence for older child age groups in 2024 was reconstructed using a synthetic cohort approach. Specifically, prevalence observed for the 2019 cohort was projected forward using empirically derived 5-year tracking coefficients describing the ratio of prevalence in the same cohort 5 years later.

**Table 2:** Baseline prevalence of weight states by age group.

Age group	Normal weight (%)	Exclusive overweight (%)	Obesity (%)	Source/derivation
5–9 years	76.00	14.80	9.20	Observed in 2024 from input UNICEF Data
10–14 years	79.40	15.58	5.02	Estimated for 2024 from 2019 cohort and 5-year tracking coefficients
15–19 years	84.75	12.41	2.84	Estimated for 2024 from 2019 cohort and 5-year tracking coefficients
Adults	45.91	35.42	18.67	Estimated for 2024 from 2019 cohort and 5-year tracking coefficients

Age-group tracking coefficients were calculated as the ratio of prevalence observed in the same cohort 5 years later to the baseline prevalence (Table 3). These coefficients quantify the persistence of overweight and obesity across consecutive age groups and were subsequently used to reconstruct age-specific prevalence and inform the calibration of the transition matrices.

**Table 3:** Estimated age-group tracking coefficients.

Weight state	Age transition	Tracking coefficient
Exclusive overweight	5–9 → 10–14	1.1459
Exclusive overweight	10–14 → 15–19	0.8220
Obesity	5–9 → 10–14	0.6272
Obesity	10–14 → 15–19	0.6311

Age tracking calculations showed that the cohort prevalence ratio for exclusive overweight was 1.1459 between the 5–9 and 10–14 age groups and 0.8220 between the 10–14 and 15–19 age groups. For obesity, the corresponding coefficients were 0.6272 and 0.6311. This indicates a more pronounced persistence of obesity between age groups with a simultaneous decrease in its prevalence in older childhood ages, while the trajectory of exclusive overweight was characterized by an increase in the transition from 5–9 to 10–14 years and a subsequent decrease by 15–19 years.

The tracking coefficient of 1.1459 for exclusive overweight between ages 5–9 and 10–14 years indicates that the prevalence of overweight increased by approximately 14.6% within the same cohort over a 5-year period. This reflects the well-documented rise in overweight prevalence during early adolescence. However, because incident transitions from normal weight to overweight cannot be identified from aggregate prevalence data, these transitions were constrained to zero in the transition matrix. Consequently, individuals in the normal-weight state were assumed to remain in that state when moving to the next age group, representing a conservative data-constrained assumption.

Table 4 shows the final transition probabilities used in the age Markov model (Equations 1–4). Each transition represents the probability of transition from a given weight state in one age group to a weight state in the next age group, which simultaneously reflects both the dynamics of the weight state and the aging of the cohort.

**Table 4:** Final transition probabilities used in the age-structured Markov model.

From state	To state	Probability
Normal 5–9	Normal 10–14	1.000
Normal 5–9	Exclusive overweight 10–14	0.000
Normal 5–9	Obesity 10–14	0.000
Exclusive overweight 5–9	Normal 10–14	0.179
Exclusive overweight 5–9	Exclusive overweight 10–14	0.821
Exclusive overweight 5–9	Obesity 10–14	0.000
Obesity 5–9	Normal 10–14	0.000
Obesity 5–9	Exclusive overweight 10–14	0.373
Obesity 5–9	Obesity 10–14	0.627
Normal 10–14	Normal 15–19	1.000
Normal 10–14	Exclusive overweight 15–19	0.000
Normal 10–14	Obesity 15–19	0.000
Exclusive overweight 10–14	Normal 15–19	0.322
Exclusive overweight 10–14	Exclusive overweight 15–19	0.678
Exclusive overweight 10–14	Obesity 15–19	0.000
Obesity 10–14	Normal 15–19	0.000
Obesity 10–14	Exclusive overweight 15–19	0.369
Obesity 10–14	Obesity 15–19	0.631
Exclusive overweight 15–19	Normal adult	0.250
Exclusive overweight 15–19	Adult exclusive overweight	0.400
Exclusive overweight 15–19	Adult obesity	0.350
Obesity 15–19	Normal adult	0.050
Obesity 15–19	Adult exclusive overweight	0.150
Obesity 15–19	Adult obesity	0.800

Because aggregated prevalence data do not allow for the identification of transitions from normal weight to overweight or obesity, these transitions were limited to zero. Similarly, the transition from obesity directly to normal weight has been set at zero as a conservative assumption.

After these structural constraints were imposed, the remaining probabilities of transitions were obtained by solving the resulting system of linear equations relating prevalence distributions across age groups. Transitions from late adolescence (15–19 years) to adulthood were determined using literature-based assumptions about maintaining a state of weight (14, 15).

### 3.1 Internal consistency of transition matrices

Table 5 presents the comparison between observed prevalence of weight states and prevalence predicted by the calibrated transition matrices. As expected from the analytical reconstruction approach, the model reproduces the observed prevalence in subsequent age groups exactly, yielding a zero discrepancy for all age transitions. This result confirms the mathematical consistency of the estimated matrices with the observed aggregate data.

**Table 5:** Observed and model-predicted values of state prevalence.

Age transition	Weight state	Observed prevalence (%)	Model-predicted prevalence (%)	Absolute difference
5–9 → 10–14	Normal weight	79.40	79.40	0.00
	Exclusive overweight	15.58	15.58	0.00
	Obesity	5.02	5.02	0.00
10–14 → 15–19	Normal weight	84.75	84.75	0.00
	Exclusive overweight	12.41	12.41	0.00
	Obesity	2.84	2.84	0.00
15–19 → Adulthood	Normal weight	45.91	45.91	0.00
	Exclusive overweight	35.42	35.42	0.00
	Obesity	18.67	18.67	0.00

Each row of the matrix satisfies two conditions:

- the sum of the transition probabilities from each initial state is equal to one;
- all transition probabilities are within the acceptable range of 0 to 1.

In addition to mathematical consistency with observed data, the transition matrices were tested for clinical plausibility. Specifically, the probability of maintaining obesity during the transition from adolescence to adulthood is higher than the probability of spontaneously transitioning from obesity to normal weight, consistent with the results of longitudinal weight tracking studies.

Thus, the transition matrices are not interpreted as directly observed individual transition probabilities. Instead, they represent one of the acceptable sets of parameters that simultaneously satisfies three conditions: reproduces the observed age-specific prevalence of weight states; is a mathematically correct probability matrix; and is consistent with published longitudinal studies of weight dynamics.

### 3.2 Financial impact of obesity

#### 3.2.1 Obesity cost structure

When calculating the financial costs, we used the diagnostic, treatment, and monitoring protocols for obese individuals prescribed in national clinical guidelines, as well as clinical recommendations for overweight people (Equations 5, 6).

Table 6 shows the cost structure and cost per child and adult per year. Calculations were made on the basis of tariffs for medical services provided within the framework of the guaranteed volume of free medical care and in the compulsory social health insurance system.

**Table 6:** Structure of healthcare resource use by model state.

Model state	Service/procedure	Frequency per year	Annual direct medical cost, *C_*s*_*, KZT (USD)
Child obesity	Pediatrician visit	2	48,365.78 (102.91)
	Endocrinologist visit	1	
	Gastroenterologist visit	1	
	Neurologist visit	1	
	Psychotherapist visit	1	
	Full pediatric laboratory panel	1	
	Abdominal ultrasound	1	
	Renal ultrasound	1	
Child overweight	Pediatrician visit	1	12,110.22 (25.77)
	Pediatric laboratory panel	0.5	
	Endocrinologist visit	*p* = 0.1	
	Ultrasound package	*p* = 0.05	
Adult obesity	GP/PHC visit	2	20,937.82 (44.55)
	Endocrinologist visit	1	
	Adult laboratory panel	1	
	Ultrasound every	0.5	
	ECG every	0.5	
Adult overweight	GP/PHC visit	1	7,364.17 (15.67)
	Adult laboratory panel	0.5	
	Endocrinologist	*p* = 0.1	
	Ultrasound	*p* = 0.05	

For the starting cohort of children aged 5–9 years in 2024, the number of children with exclusive overweight was 296,250, and with obesity-184,155. So according to Formula 7 the corresponding annual direct medical cost is 3,587,652,675.00 KZT (7,633,304 USD) for child exclusive overweight and 8,906,800,215.90 KZT (18,950,639 USD) for child obesity, and the total annual direct medical cost is 12,494,452,890.90 KZT (26,583,942 USD).

Table 7 presents the discounted lifetime direct medical cost per child estimated by the Markov model. These values represent the expected present value of future healthcare expenditures associated with different initial weight states at ages 5–9 years, calculated using a 3% annual discount rate (Equations 7–9).

**Table 7:** Discounted lifetime direct medical cost per child.

Starting state	*LC*, KZT (USD)
Child with exclusive overweight at ages 5–9	258,497.64 (549.99)
Child with obesity at ages 5–9	547,147.04 (1164.14)
Average per child in the whole 2024 cohort aged 5–9	96,996.26 (206.37)

The average lifetime cost per child in the baseline cohort [96,996.26 KZT (206.37 USD)] represents the expected present value of future healthcare expenditures associated with childhood overweight and obesity, weighted by the prevalence of each weight state in the population. According to Equation 10, for the national starting cohort of 2,001,691 children aged 5–9 in 2024, the estimated discounted national total lifetime burden is 194,156,540,675 KZT (413,099,022.7 USD).

#### 3.2.2 Projected cumulative direct medical burden among children aged 5–9 years, 2024–2035

The purpose of this analysis was to estimate the cumulative direct medical expenditure associated with childhood overweight and obesity over the medium-term horizon of 2024–2035. While the lifetime burden analysis reflects the long-term economic consequences of excess weight in a single cohort of children, this additional analysis quantifies the expected healthcare expenditure attributable to overweight and obesity among children aged 5–9 years in the population during the next decade (Equations 11–12). This approach provides an estimate of the short- and medium-term financial pressure on the healthcare system.

The decline in annual cost values over time (Table 8) is driven by the application of discounting of a 3% rather than changes in obesity prevalence. In this analysis, prevalence was assumed to remain constant at the 2024 level. Therefore, the observed decrease reflects the lower present value assigned to future costs, rather than a reduction in the underlying financial impact.

**Table 8:** Cumulative discounted direct medical burden for 2024–2035, KZT bn (USD mn).

Year	Annual exclusive overweight	Annual obesity	Annual total	Cumulative exclusive overweight	Cumulative obesity	Cumulative total
2024	3.60 (7.67)	8.95 (19.07)	12.55 (26.71)	3.60 (7.67)	8.95 (19.07)	12.55 (26.71)
2025	3.50 (7.46)	8.69 (18.50)	12.19 (25.94)	7.10 (15.13)	17.64 (37.57)	24.74 (52.65)
2026	3.39 (7.22)	8.44 (17.96)	11.83 (25.18)	10.49 (22.35)	26.08 (55.53)	36.58 (77.82)
2027	3.30 (7.03)	8.19 (17.42)	11.49 (24.45)	13.79 (29.38)	34.27 (72.95)	48.07 (102.27)
2028	3.20 (6.81)	7.95 (16.92)	11.15 (23.74)	16.99 (36.19)	42.22 (89.87)	59.22 (126.00)
2029	3.11 (6.62)	7.72 (16.44)	10.83 (23.04)	20.10 (42.81)	49.94 (106.31)	70.05 (149.04)
2030	3.02 (6.43)	7.49 (15.95)	10.51 (22.38)	23.12 (49.24)	57.43 (122.26)	80.57 (171.42)
2031	2.93 (6.24)	7.28 (15.48)	10.21 (21.72)	26.05 (55.48)	64.71 (137.74)	90.77 (193.13)
2032	2.84 (6.05)	7.07 (15.04)	9.91 (21.09)	28.89 (61.53)	71.78 (152.78)	100.68 (214.22)
2033	2.76 (5.88)	6.86 (14.60)	9.62 (20.46)	31.65 (67.41)	78.64 (167.38)	110.31 (234.69)
2034	2.68 (5.71)	6.66 (14.17)	9.34 (19.87)	34.33 (73.12)	85.30 (181.55)	119.65 (254.57)
2035	2.60 (5.53)	6.47 (13.77)	9.07 (19.29)	36.93 (78.65)	91.79 (195.22)	128.72 (273.87)
Annual values are shown in present value terms (discounted at 3%)						

On the horizon of 2024–2035, the accumulated discounted direct medical cost, calculated at a rate of 3% and assuming a constant annual burden at the 2024 level, steadily increased due to the summation of discounted annual costs. In 2024, the annual direct medical cost amounted to 3.60 billion tenge (7.67 USD mn) for child overweight and 8.95 billion tenge (19.07 USD mn) for child obesity, a total of 12.55 billion tenge (26.71 USD mn). By 2035, the cumulative discounted cost reached 36.93 billion tenge (78.65 USD mn) for overweight and 91.79 billion tenge (195.22 USD mn) for obesity, which amounted to a total of 128.72 billion tenge (273.87 USD mn). Thus, during the 12-year period under review, about 71.3% of the accumulated direct medical expenditure was accounted for by childhood obesity, which confirms its dominant contribution to the total short- and medium-term health system costs associated with childhood overweight.

#### 3.2.3 Sensitivity analysis results

The discounted national lifetime burden estimate for the 2024 cohort of children aged 5–9 years was used as a baseline of 194.16 billion KZT and the average discounted lifetime cost per child of 96,996.26 KZT (Table 7). The baseline values are taken from a consistent version of the model.

The following parameters were included in the analysis:

- baseline prevalence of exclusive overweight: ±20%;
- baseline prevalence of obesity: ±20%;
- discounted lifetime cost per child with exclusive overweight: ±20%;
- discounted lifetime cost per child with obesity: ±20%;
- cohort size of children aged 5–9 years: ±10%.

For each scenario, the average discounted lifetime cost per child and discounted national lifetime economic impact were recalculated (Table 9). This approach makes it possible to determine which parameters have the greatest impact on the final assessment of the financial burden.

**Table 9:** One-way sensitivity analysis.

Parameter varied	Range	Low-case national lifetime burden, KZT bn (USD mn)	High-case national lifetime burden, KZT bn (USD mn)	Change vs. base, low	Change vs. base, high
Baseline prevalence: exclusive overweight	±20%	180.68 (384.4)	207.64 (441.8)	−6.94%	+6.94%
Baseline prevalence: obesity	±20%	168.96 (359.5)	219.36 (466.7)	−12.98%	+12.98%
Discounted lifetime cost per child with exclusive overweight	±20%	180.68 (384.4)	207.64 (441.8)	−6.94%	+6.94%
Discounted lifetime cost per child with obesity	±20%	168.96 (359.5)	219.36 (466.7)	−12.98%	+12.98%
Cohort size (children aged 5–9 years)	±10%	174.74 (371.8)	213.58 (454.4)	−10.00%	+10.00%

In a univariate deterministic sensitivity analysis, obesity-related parameters had the greatest impact on discounted national lifetime burden. A ±20% change in baseline prevalence of obesity and discounted lifetime cost per child with obesity resulted in a ±12.98% change in the final burden estimate. For the parameters of exclusive overweight, the corresponding change was ±6.94%. A ±10% change in the cohort of children aged 5–9 years caused a proportional change in national lifetime burden of ±10.0%. Thus, the results of the model turned out to be the most sensitive to the prevalence of obesity in children and to the lifetime costs associated with obesity.

#### 3.2.4 Structural scenario analysis

Results of the structural scenario analysis are presented in Table 10. The base-case specification produced a discounted national lifetime burden of 194.16 billion KZT for the 2024 cohort of children aged 5–9 years.

**Table 10:** Structural scenario analysis evaluating the impact of alternative modeling assumptions on discounted lifetime economic burden of childhood overweight and obesity in Kazakhstan.

Scenario	Structural assumption tested	Discounted lifetime cost per child, KZT (USD)	National lifetime burden, KZT bn (USD mn)	Change vs. base
Scenario A—base case	Normal → OW = 0; Normal → OB = 0; OB → Normal = 0; constant adult costs	96,996 (206.37)	194.16 (413.10)	Reference
Scenario B—less restrictive transitions	Small incident transitions from normal weight allowed	102,071 (217.17)	204.26 (434.60)	+5.2%
Scenario C—age-varying adult costs	Adult overweight and obesity costs increase with age	110,199 (234.47)	220.57 (469.30)	+13.6%

Relaxing the restriction on incident transitions (Scenario B) resulted in a modest increase in the estimated cost, reflecting the possibility that some children with normal weight develop excess weight during adolescence. Under this specification, the estimated national lifetime healthcare costs increased to 204.26 billion KZT, representing an increase of approximately 5.2% relative to the base case.

Introducing age-varying adult medical costs (Scenario C) produced a larger effect on model outcomes. When adult obesity-related costs were allowed to increase with age, the discounted national lifetime burden increased to 220.57 billion KZT, corresponding to an increase of approximately 13.6% compared with the base case.

Overall, the scenario analysis indicates that the base-case model likely provides a conservative estimate of the long-term economic impact of childhood exclusive overweight and obesity. In particular, assumptions regarding adult cost profiles have a substantial influence on the magnitude of lifetime medical expenditures.

#### 3.2.5 Policy scenario analysis

The policy scenario analysis evaluated the potential economic impact of reductions in childhood obesity prevalence relative to the base-case model, which assumes that the prevalence of exclusive overweight and obesity remains constant at the 2024 level.

Reducing childhood obesity prevalence produced measurable reductions in the projected lifetime economic expenditure associated with excess weight (Table 11). In the scenario assuming a 5% reduction in childhood obesity prevalence, the discounted lifetime cost per child decreased from 96,996 KZT (206.37 USD) in the base case to 92,300 KZT (196.38 USD), corresponding to a reduction in the national lifetime economic burden from 194.16 billion KZT (413.1 USD mn) to 184.84 billion KZT (392.34 USD mn).

**Table 11:** Projected economic burden under childhood obesity prevention scenarios.

Scenario	Assumption	Discounted lifetime cost per child, KZT (USD)	National lifetime burden, KZT bn (USD mn)	Change vs. base
Base case	Prevalence remains at 2024 level	96,996 (206.37)	194.16 (413.10)	—
5% reduction scenario	Childhood obesity prevalence −5%	92,300 (196.38)	184.84 (393.30)	−4.8%
10% reduction scenario	Childhood obesity prevalence −10%	87,800 (186.81)	175.91 (374.30)	−9.4%

A larger effect was observed in the 10% reduction scenario. Under this assumption, the discounted lifetime cost per child decreased to 87,800 KZT (186.81 USD), and the national lifetime medical cost declined to 175.91 billion KZT (374.27 USD mn). This represents a reduction of approximately 9.4% relative to the base-case estimate.

Overall, the results indicate that even modest reductions in childhood obesity prevalence can lead to substantial long-term savings in healthcare expenditure. The magnitude of these savings increases proportionally with the scale of obesity prevention.

## 4 Discussion

This study presents the first comprehensive assessment of the economic impact of childhood exclusive overweight and obesity in the Republic of Kazakhstan using a dynamic age-structured Markov model. The results demonstrate that even assuming a constant prevalence of exclusive overweight and obesity at 2024 levels, these conditions create significant short- and long-term financial pressure on the healthcare system.

The key result of the research is an estimate of the lifetime healthcare costs for a cohort of children aged 5–9 years. The expected present value of medical expenses per child was 96,996.26 tenge (USD 206.37), and the cumulative lifetime burden for the national cohort was 194 billion KZT (USD 413 million). These values reflect the cumulative effect of exclusive overweight and obese children transitioning to adulthood, where medical costs are typically higher and associated with the development of chronic non-communicable diseases. An important addition to the lifetime assessment was the modeling of the short- and medium-term cumulative lifetime healthcare expenditure for the period 2024–2035. It was shown that even with a fixed obesity prevalence, annual costs accumulate and reach 128.7 billion KZT (273.9 million USD) over a 12-year period, with obesity accounting for over 70% of these costs. This emphasizes that the economic impact of the problem manifests itself not only in the long term but also in the coming years, creating ongoing pressure on the healthcare system.

Our findings align with a recent systematic review of obesity costs in LMICs, which reported annual direct costs ranging from USD 544 million to USD 12.56 billion (20). Comparisons with other middle-income countries show that the economic burden of obesity varies widely but is significant across the board. In Brazil, annual hospitalization costs for patients with class III obesity reach $1,840 per person, and each 1% increase in obesity prevalence is associated with additional hospitalization costs of approximately $10 million. In Thailand, as in Kazakhstan, direct medical costs of obesity are estimated to be higher than indirect ones, which may reflect labor market characteristics in countries with high informal employment rates. In Mexico, costs of obesity-related complications consume up to 87% of the programmable healthcare budget, highlighting the threat to the financial sustainability of the system. These data are consistent with our findings that childhood obesity creates long-term pressure on the healthcare system in Kazakhstan and support the need for preventive interventions early in life.

A comparison with international data shows that our estimates of direct medical costs fall at the lower end of the range, which can be attributed to lower per capita healthcare expenditures in Kazakhstan and the model's conservative assumptions. In the meta-analysis by Ling et al. (7), the average annual additional costs per child with obesity amounted to 307.72 USD, which significantly exceeds our annual costs of managing obesity. Kazakhstan has a pre-dominantly publicly financed healthcare system, in which a substantial share of essential medical services is provided through the guaranteed volume of free medical care and the compulsory social health insurance system. Consequently, healthcare expenditures estimated from nationally regulated tariffs may be substantially lower than costs reported in high-income countries, particularly the United States and some European settings. Differences in provider reimbursement, labor costs, diagnostic intensity, access to specialist care, and patterns of healthcare utilization may therefore partly explain why the estimated direct medical costs of childhood obesity in Kazakhstan fall below many international estimates. Importantly, lower monetary expenditure should not be interpreted as indicating a proportionally lower health burden. Rather, it reflects differences in healthcare prices, financing arrangements, service availability, and resource use across health systems. Therefore, direct cross-country comparisons of obesity-related costs should be interpreted cautiously.

However, even these higher international estimates of direct costs do not reflect the full economic burden of obesity, as they do not account for loss of quality of life and pre-mature mortality. An alternative approach, based on the value of a statistical life (VSL), allows us to estimate society's willingness to pay to reduce the risk of death from obesity. In a study (21), this estimate for Kazakhstan ranged from 39 USD million (using DALYs) to 60.6 USD billion (using the total number of deaths).

Sensitivity analysis results showed that the model was most sensitive to parameters related to obesity, particularly its prevalence and lifetime costs. A ±20% change in these parameters resulted in a change in the overall cost of almost ±13%, while parameters related to exclusive overweight had a significantly smaller effect.

The economic burden was largely concentrated on obesity, not solely overweight. At the individual level, the estimated annual direct medical costs of childhood obesity were approximately four times greater than the corresponding costs of solely overweight children. At the population level, childhood obesity accounted for approximately 71.3% of the total discounted direct medical burden for the period 2024–2035. This concentration of costs once again demonstrates that obesity, not solely overweight, is the primary cause of direct medical burden and highlights the potential economic value of preventive strategies that successfully reduce the prevalence of childhood obesity.

Structural scenario analysis showed that the baseline model, based on conservative assumptions, likely underestimates the true financial burden. Relaxing restrictions on state transitions leads to increased estimates, and accounting for age-related increases in medical costs in adulthood increases the lifetime healthcare expenditure by 13.6%. This suggests that the actual healthcare costs associated with obesity may be significantly higher than the baseline estimates.

The results of the policy scenario analysis are particularly significant from a policy planning perspective. Our projections suggest that even a hypothetical 5% reduction in childhood obesity prevalence could lead to a reduction in the lifetime economic burden by approximately 5%, and a 10% reduction by approximately 10%. While these figures are derived from modeling exercises and do not represent empirical evidence for specific interventions, they illustrate the potential economic returns from achieving meaningful reductions in obesity prevalence. These findings underscore that investments in effective prevention strategies-such as school-based nutrition programs, physical activity promotion, and regulatory measures targeting obesogenic environments-could yield substantial long-term savings if they successfully reduce obesity rates. However, the actual magnitude of savings would depend on the effectiveness of the specific interventions implemented.

From a methodological perspective, an important contribution of the study is the use of a synthetic cohort approach to reconstruct transition probabilities based on repeated cross-sectional data. In the absence of longitudinal observations, this approach allows for the creation of an internally consistent transition system and the reproduction of observed prevalence dynamics. Furthermore, the introduction of structural constraints on transitions (for example, excluding transitions from normal body weight directly to obesity) ensures the epidemiological plausibility of the model, although it makes the estimates more conservative.

Despite the obtained results, the study has several limitations. An important structural limitation of our base-case model is the restriction on transitions from normal weight to exclusive overweight or obesity. This assumption, while necessary due to data constraints, reduces epidemiological realism. By excluding the possibility that children with normal weight at ages 5–9 may develop excess weight later in life, the model provides a conservative estimate of the lifetime burden. The structural scenario analysis confirms this: when small incident transitions from normal weight were allowed (Scenario B), the estimated national lifetime cost increased by 5.2%. This suggests that the true economic impact may be higher than our base-case estimates, and that our findings should be interpreted as a lower-bound reference for policy planning.

The use of aggregated prevalence data does not allow for direct observation of individual trajectories of transitions between states, requiring assumptions when calibrating transition matrices. Furthermore, annual medical costs were estimated using a normative approach and may not fully reflect actual consumption of medical services. Third, the baseline model assumed a constant obesity prevalence, which rules out possible future changes in the epidemiological situation. Finally, data on age-related cost dynamics in the adult population were obtained indirectly and require further validation using national data.

Another important limitation is the exclusion of mortality and disease progression from the model. The lifetime cost estimates assume that all individuals survive to the end of the model horizon, regardless of their weight state. This assumption may lead to overestimation of lifetime healthcare costs, as individuals with obesity in the model continue to accumulate costs for a full lifespan, whereas in reality they may experience pre-mature mortality. Conversely, by not modeling the progression of obesity-related complications (e.g., type 2 diabetes, cardiovascular disease, metabolic disorders), the model may underestimate the true direct medical costs, as these conditions require substantial additional healthcare resources. The net direction of bias is therefore uncertain and depends on the relative magnitude of these two countervailing effects. Future research incorporating nationally representative mortality and morbidity data would be valuable to refine these estimates.

A further limitation of this study is the exclusion of indirect costs and broader societal impacts. Our estimates reflect only direct medical expenditures incurred by the healthcare system and do not account for productivity losses due to absenteeism, presenteeism, disability, or pre-mature mortality associated with obesity. They also do not capture the intangible costs of reduced quality of life, psychological distress, social stigma, or the financial burden on families and caregivers. Evidence from other settings suggests that indirect costs can constitute a substantial proportion of the total economic expenditure of obesity, sometimes exceeding direct medical costs (22). Therefore, the estimates presented here are likely to be conservative and should be interpreted as a lower-bound reference for the economic burden of childhood obesity in Kazakhstan. Future research incorporating indirect costs and quality-of-life impacts would provide a more comprehensive assessment of the societal cost.

At the same time, the study has several significant advantages. Among them, it is worth noting that the model combines demographic and epidemiological dynamics, allowing for the assessment of the full life cycle of medical burden. Also, for the first time in Kazakhstan, consistent estimates of both lifetime and short-term expenditure were obtained, increasing the practical significance of the results. Thirdly, the scenario analysis conducted allows us to assess the impact of both parametric and structural uncertainty, as well as the potential effect of preventive interventions.

Given that obesity accounts for the majority of the healthcare resource costs and increases with age, early interventions in childhood can deliver the greatest economic returns. This includes well-established measures aimed at promoting healthy eating habits, increasing physical activity, and limiting factors that contribute to obesity.

The findings also have implications beyond Kazakhstan. Many countries with constrained health information systems lack longitudinal data linking childhood weight trajectories to healthcare utilization and long-term costs. Our study demonstrates that routinely available cross-sectional prevalence data, national demographic statistics, clinical protocols, and healthcare tariffs can be integrated within a synthetic cohort modeling framework to generate policy-relevant estimates of the long-term economic burden of childhood obesity. Although absolute monetary costs will differ substantially across health systems, the concentration of healthcare expenditure among children with obesity and the accumulation of these costs across the life course are likely to be relevant to other resource-constrained settings experiencing rapid nutritional and epidemiological transitions. The approach may therefore provide a practical framework for countries where longitudinal cohorts and detailed claims databases are unavailable, while highlighting the economic rationale for investing in childhood obesity prevention before substantial downstream healthcare costs accumulate.

## 5 Conclusion

In this study, childhood obesity in Kazakhstan is viewed not merely as a clinical problem, but as a structural factor determining long-term healthcare costs. The results reveal three policy-relevant findings: obesity accounts for the lion's share of the economic impact, justifying targeted interventions; the nearly linear dose-response relationship between prevalence and costs confirms the need for prevention even with moderate effect sizes; and the baseline estimates are likely conservative, meaning that inaction entails higher costs than assumed. From a methodological standpoint, the synthetic cohort approach offers a reproducible framework for studying the burden of disease under conditions of limited data. Ultimately, delaying prevention will not reduce future costs; it will merely postpone them, while allowing the underlying epidemiological factors to persist.

## Data Availability

The original contributions presented in the study are included in the article/Supplementary material, further inquiries can be directed to the corresponding author.
