# Supplementary material for: Two horizons, one burden: estimating lifetime and decade-long medical costs of childhood obesity in Kazakhstan
Source: Front Public Health. 2026 Oct 2;14:1853931. doi: 10.3389/fpubh.2026.1853931 (PMC13672551; doi:10.3389/fpubh.2026.1853931)
Supplement: Supplementary file 1 [file Table_1.docx]

**SUPPLEMENTARY APPENDIX A: DETAILED COSTING ASSUMPTIONS**

Costs were estimated using a normative costing approach based on national clinical protocols and relevant tariffs for outpatient care, laboratory investigations, diagnostic procedures, and follow-up management. The following clinical protocols were used as the basis for resource utilization:

- *Clinical Protocol for Diagnosis and Treatment of Obesity in Children and Adolescents*. Approved by the Joint Commission on the Quality of Medical Services of the Ministry of Health of the Republic of Kazakhstan on August 18, 2017, Protocol No. 26.
- *Clinical Protocol for Diagnosis and Treatment of Obesity in Adults*. Approved by the Joint Commission on the Quality of Medical Services of the Ministry of Health of the Republic of Kazakhstan on December 7, 2023, Protocol No. 196.

Tariffs were obtained from the official schedule approved by the Ministry of Health of the Republic of Kazakhstan (Order No. 12 of April 5, 2024, amending Order No. ҚР ДСМ-170/2020 of the Acting Minister of Health of the Republic of Kazakhstan dated October 30, 2020 "On approval of tariffs for medical services provided within the framework of the guaranteed volume of free medical care and in the compulsory social health insurance system").

All costs were calculated in 2024 prices and are expressed in Kazakhstan tenge (KZT).

**Table A1. Annual healthcare costs for children with obesity (based on compulsory social health insurance tariffs)**

| Service | Frequency per year | Tariff (KZT) | Annual cost (KZT) |
| --- | --- | --- | --- |
| **Outpatient consultations** |  |  |  |
| Pediatrician visit | 2 | 2,487.71 | 4,975.42 |
| Endocrinologist visit | 1 | 4,097.75 | 4,097.75 |
| Gastroenterologist visit | 1 | 4,266.15 | 4,266.15 |
| Neurologist visit | 1 | 4,097.75 | 4,097.75 |
| Psychotherapist visit | 1 | 4,029.28 | 4,029.28 |
| **Laboratory investigations** |  |  |  |
| Complete blood count | 1 | 725.86 | 725.86 |
| **Biochemistry panel** |  |  |  |
| — Glucose | 1 | 634.88 | 634.88 |
| — Oral glucose tolerance test (OGTT) | 1 | 1,700.00 | 1,700.00 |
| **Lipid profile** |  |  |  |
| — Total cholesterol | 1 | 1,700.00 | 1,700.00 |
| — HDL cholesterol | 1 | 1,729.02 | 1,729.02 |
| — LDL cholesterol | 1 | 506.73 | 506.73 |
| — Triglycerides | 1 | 3,347.88 | 3,347.88 |
| **Hormonal assays** |  |  |  |
| — ACTH | 1 | 2,875.87 | 2,875.87 |
| — Cortisol | 1 | 2,328.32 | 2,328.32 |
| — TSH | 1 | 1,935.36 | 1,935.36 |
| **Imaging** |  |  |  |
| Abdominal ultrasound (liver, gallbladder) | 1 | 6,309.99 | 6,309.99 |
| Renal ultrasound | 1 | 3,105.52 | 3,105.52 |
|  |  | **Total** | **48,365.78** |

**Additional clinical considerations for children with obesity:**

- 60.7% of children with obesity have insulin resistance
- 16.9% of children with obesity have hypertension

**Table A2. Annual healthcare costs for adults with obesity (based on compulsory social health insurance tariffs)**

| Service | Frequency per year | Tariff (KZT) | Annual cost (KZT) |
| --- | --- | --- | --- |
| **Outpatient consultations** |  |  |  |
| General practitioner/PHC visit | 2 | 1,326.44 | 2,652.88 |
| Endocrinologist consultation | 1 | 4,097.75 | 4,097.75 |
| **Laboratory investigations** |  |  |  |
| **Carbohydrate metabolism** |  |  |  |
| — Fasting glucose | 1 | 725.86 | 725.86 |
| — HbA1c | 1 | 527.32 | 527.32 |
| **Lipid profile** |  |  |  |
| — Total cholesterol | 1 | 634.88 | 634.88 |
| — HDL cholesterol | 1 | 1,700.00 | 1,700.00 |
| — LDL cholesterol | 1 | 1,700.00 | 1,700.00 |
| — Triglycerides | 1 | 1,729.02 | 1,729.02 |
| **Liver function tests** |  |  |  |
| — ALT | 1 | 717.15 | 717.15 |
| — AST | 1 | 562.37 | 562.37 |
| **Endocrine screening** |  |  |  |
| — TSH | 1 | 2,328.32 | 2,328.32 |
| **Imaging and diagnostics** |  |  |  |
| Ultrasound | 0.5 | 6,309.99 | 3,155.00 |
| ECG | 0.5 | 814.55 | 407.00 |
|  |  | **Total** | **20,937.82** |

**Table A3. Annual healthcare costs for overweight individuals (children and adults)**

A normative management pathway for overweight was constructed based on international clinical guidelines recommending regular assessment of body weight and cardiometabolic risk, provision of behavioural interventions, periodic laboratory screening, and referral to specialists when clinically indicated. These components are consistently emphasised across NICE, Canadian Adult Obesity Clinical Practice Guidelines, VA/DoD obesity guidance, and Endocrine Society recommendations.

| Service | Frequency per year | Tariff (KZT) | Annual cost (KZT) |
| --- | --- | --- | --- |
| **Children with overweight** |  |  |  |
| Pediatrician visit | 1 | 2,487.71 | 2,487.71 |
| Laboratory panel | 0.5 | 15,200.00* | 7,600.00 |
| Endocrinologist consultation (p=0.1) | 0.1 | 4,097.75 | 409.78 |
| Ultrasound package (p=0.05) | 0.05 | 6,309.99 | 315.50 |
| *Other services* |  |  | 1,297.23 |
|  |  | **Total** | **12,110.22** |
| **Adults with overweight** |  |  |  |
| General practitioner/PHC visit | 1 | 1,326.44 | 1,326.44 |
| Laboratory panel | 0.5 | 10,800.00* | 5,400.00 |
| Endocrinologist consultation (p=0.1) | 0.1 | 4,097.75 | 409.78 |
| Ultrasound (p=0.05) | 0.05 | 6,309.99 | 315.50 |
| *Other services* |  |  | -87.55 |
|  |  | **Total** | **7,364.17** |

*Note: Laboratory panel costs represent composite tariff estimates based on the sum of individual laboratory tests. p = probability of receiving the service. PHC = primary healthcare center.*

**Summary Table: Annual direct medical costs by model state**

| Model state | Annual direct medical cost (KZT) | Annual direct medical cost (USD)* |
| --- | --- | --- |
| Child obesity | 48,365.78 | 102.91 |
| Child overweight | 12,110.22 | 25.77 |
| Adult obesity | 20,937.82 | 44.55 |
| Adult overweight | 7,364.17 | 15.67 |

*Converted at the 2024 average exchange rate of 470 KZT/USD.

**Notes for Supplementary Appendix A:**

1. All costs are expressed in 2024 Kazakhstan tenge (KZT) and reflect **payer costs** from the healthcare system perspective (government expenditures within the guaranteed volume of free medical care and the compulsory social health insurance system).
2. Frequencies and service lists are based on national clinical protocols for the management of overweight and obesity in children and adults (Protocols No. 26, 2017 and No. 196, 2023).
3. Unit tariffs are from Order No. 12 of the Ministry of Health of the Republic of Kazakhstan (April 5, 2024).
4. No inflation adjustments were applied, as all costs are expressed in constant 2024 prices.
5. The costs for overweight individuals include probabilistic components (p-values) reflecting the proportion of patients expected to receive specialist consultations or imaging based on clinical judgement and guideline recommendations.
6. "Other services" in the overweight categories represent residual costs covering additional consultations, minor procedures, or patient education not separately itemized in the tariff schedule.
